# Prevention and Management of Tumor Lysis Syndrome in Adults With Malignancy

**Published:** 2013-03-01

**Authors:** Jessica Sarno

## Abstract

Tumor lysis syndrome (TLS), an oncologic emergency that typically occurs after the treatment of a malignancy with chemotherapy and/or radiotherapy, is the result of extreme tumor cell lysis with the release of intracellular potassium, nucleic acids, and phosphorus into the systemic circulation. Tumor lysis syndrome occurs most often after administration of cytotoxic therapy in patients with high-grade lymphomas and acute lymphoblastic leukemia, but it can also occur spontaneously in tumor types that have a high proliferative rate and/or a large tumor burden. The metabolic disturbances of TLS include hyperkalemia, hyperphosphatemia, secondary hypocalcemia, hyperuricemia, and acute renal failure. The most important treatment for TLS is prevention. The mainstays of TLS prevention include aggressive hydration, control of hyperuricemia with allopurinol and rasburicase treatment, and close monitoring of electrolyte abnormalities. It is crucial for clinicians to prevent, detect, and treat TLS early to prevent life-threatening complications such as acute renal failure, cardiac dysrhythmia, and seizures. The purpose of this article is to explain the pathophysiology of TLS, identify patients at risk for TLS, and detail strategies for prevention and management of this oncologic emergency.

In a malignancy with a high proliferative rate, large tumor burden, or high sensitivity to treatment, initiation of chemotherapy and/or radiation therapy can induce rapid lysis of tumor cells resulting in tumor lysis syndrome (TLS; Larson & Pui, 2012a). Tumor lysis syndrome is most often brought about by chemotherapy and radiation, although intrathecal chemotherapy, as well as biological agents such as rituximab (Rituxan), can also induce this oncologic emergency (McCurdy & Shanholtz, 2012). Other biological agents that have been reported to cause TLS include cetuximab (Erbitux), sunitinib (Sutent), and bortezomib (Velcade; Krishnan, D’Silva, & Al-Janadi, 2008). Although TLS is most commonly detected after the administration of cytotoxic therapy, it can occur spontaneously prior to therapy in rapidly proliferating tumors (McCurdy & Shanholtz, 2012). The death of tumor cells results in a release of intracellular contents such as potassium, phosphorus, and nucleic acids into the systemic circulation. The release of intracellular contents results in electrolyte disturbances: hyperkalemia, hyperphosphatemia, hyperuricemia, and secondary hypocalcemia, as well as organ dysfunction such as acute renal failure (Pession et al., 2011).

Tumor lysis syndrome is most frequently associated with acute lymphoblastic leukemia (ALL), Burkitt’s lymphoma, and acute myeloid leukemia (AML), but it has also been associated with other hematologic malignancies such as multiple myeloma, chronic lymphocytic leukemia, and low-grade and intermediate-grade non-Hodgkin lymphoma (Cairo, Coiffier, Reiter, & Younes, 2011). Tumor lysis syndrome is most common in hematologic malignancies, yet solid tumors with high proliferative rates like testicular cancer and small-cell lung cancer may also cause TLS (Cairo & Bishop, 2004). The incidence of TLS varies greatly and depends on the type of neoplasm and treatment administered (Burghi, Berrutti, & Manzanares, 2011). In elderly patients the diagnosis of TLS is more serious secondary to preexisting comorbidities such as chronic renal insufficiency and cardiac disease (Burghi, Berrutti, & Manzanares, 2011). The identification of patients, at risk for development of TLS is the most critical aspect of management so that prophylactic measures can be taken prior to the initiation of cancer treatment.

## Risk Factors

The risk for TLS is derived from many individual factors, including age, type and stage of cancer, lactate dehydrogenase level (LDH), white blood cell (WBC) count, baseline renal function, and patient comorbidities (Cairo et al., 2010). In order to address risk stratification for TLS, an expert panel of oncologists met in Paris in 2008 to develop criteria to identify patients at low, intermediate, and high risk for TLS (Table 1). Low-risk disease (LRD) was defined as a less than 1% risk of developing TLS, intermediate-risk disease (IRD) was defined as a 1% to 5% risk of developing TLS, and high-risk disease (HRD) was defined as a greater than 5% risk of developing TLS.

**Table 1:**
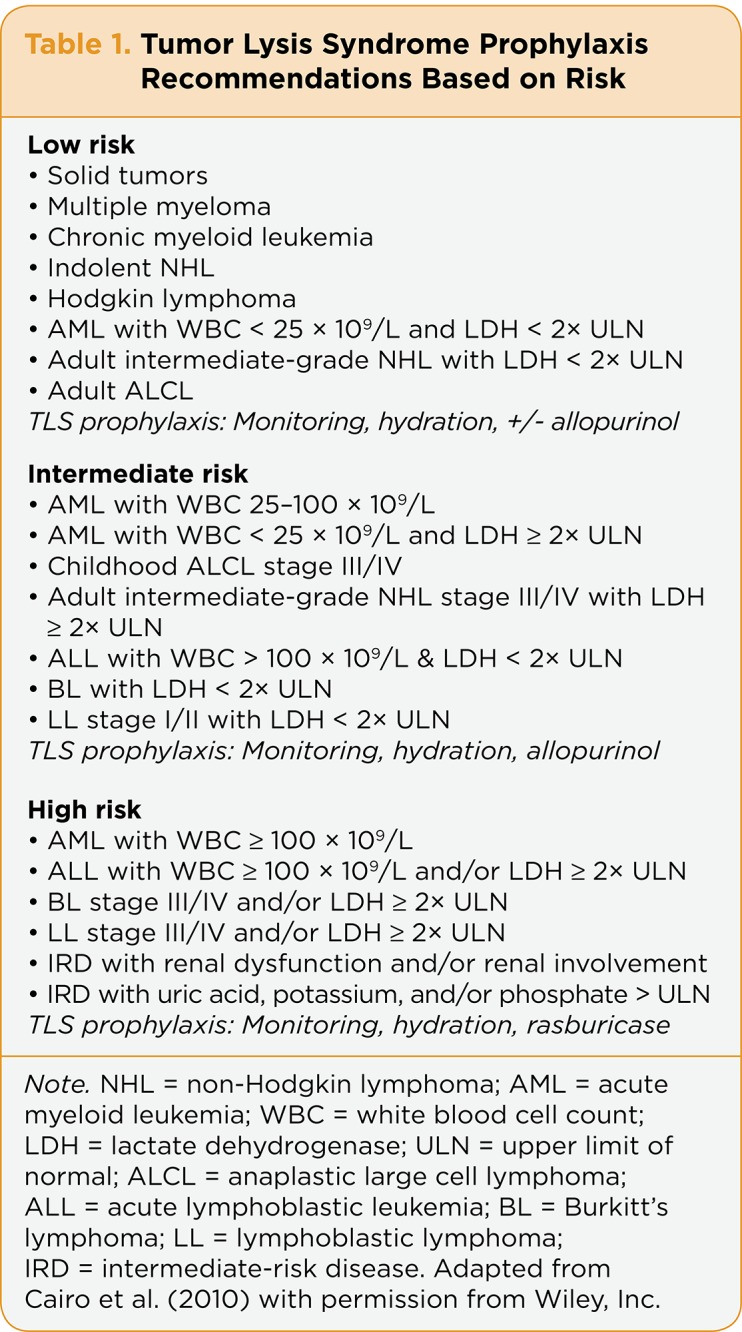
Table 1. Tumor Lysis Syndrome Prophylaxis Recommendations Based on Risk

Risk evaluation was first based on identifying patients with laboratory TLS (LTLS). Patients must have two or more abnormalities of elevated uric acid, elevated potassium, or elevated phosphate levels to be diagnosed with LTLS. Second, malignancies (both solid tumor and hematologic) were classified as LRD, IRD, or HRD. Most solid tumors were classified as LRD, but bulky tumors that are sensitive to chemotherapy, such as germ-cell tumors and small-cell tumors, were graded as IRD. In addition, multiple myeloma was graded as LRD. Risk identification for TLS in acute leukemias and lymphomas was assessed based on WBC count and LDH levels. Burkitt’s lymphoma was always considered HRD (Cairo et al., 2010).

Finally, patients were evaluated by age, disease bulk, WBC count, and LDH level, and adjustments were made based on renal function (Cairo et al., 2010). For example, patients with LRD are considered at intermediate risk if renal dysfunction or renal involvement is present. In addition, patients with IRD and normal renal function would be considered at high risk for TLS if hyperuricemia, hyperphosphatemia, or hyperkalemia is evident (Cairo et al., 2010). This classification system can guide clinicians in administering the appropriate TLS prophylaxis for their patients at varying levels of risk (see Table 1).

## Diagnosis

Tumor lysis syndrome can be broken down into two types: the previously mentioned LTLS or clinical tumor lysis syndrome (CTLS). The Cairo-Bishop definition of LTLS provides specific criteria for LTLS and is defined as 25% above/below normal for two or more levels of uric acid, potassium, phosphate, and calcium within 3 days before or 7 days after administration of chemotherapy (Cairo & Bishop, 2004). The definition of CTLS includes a diagnosis of LTLS in addition to the presence of either a serum creatinine level 1 to 5 times greater than the upper limit of normal, cardiac arrhythmia/sudden death, or seizure (Cairo & Bishop, 2004). In addition, it must be noted that the rise in creatinine cannot be attributed to other causes such as the administration of a nephrotoxic medication. The Cairo-Bishop grading system for TLS (see Table 2) includes definitions of TLS, LTLS, and CTLS and the clinical manifestations (renal, neurologic, and cardiac) that dictate the grade of TLS (Cairo & Bishop, 2004).

**Table 2:**
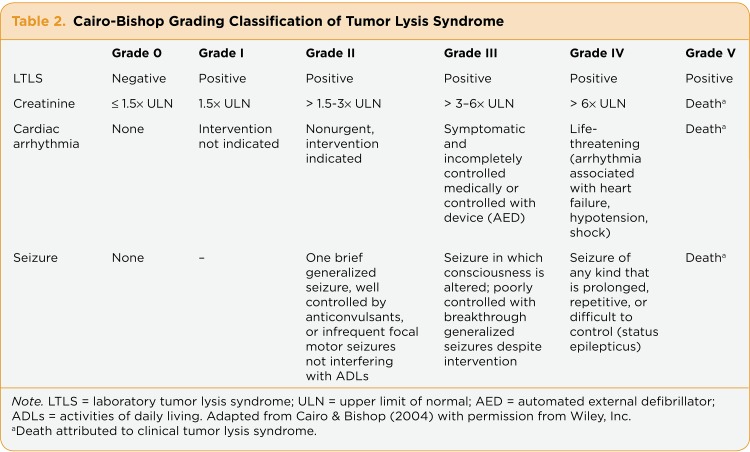
Table 2.Cairo-Bishop Grading Classification of Tumor Lysis Syndrome

## Clinical Manifestations

The clinical manifestations of TLS can be attributed to the individual metabolic disturbances that characterize this syndrome: hyperuricemia, hyperkalemia, and hyperphosphatemia. Hyperuricemia is secondary to the catabolism of purine nucleic acids (McCurdy & Shanholtz, 2012). Uric acid is cleared through the kidneys (Cairo & Bishop, 2004). When hyperuricemia (uric acid level ≥ 7.5 mg/dL) occurs, patients may experience nausea, vomiting, diarrhea, and anorexia as well as acute renal failure (McCurdy & Shanholtz, 2012). Renal failure results from hyperuricemia secondary to uric acid crystal precipitation in the renal tubules (McCurdy & Shanholtz, 2012). At times, flank pain can occur if there is uric acid ureteral stone formation (Larson & Pui, 2012a). In patients experiencing hyperuricemia, urinalysis may reveal uric acid crystals (Larson & Pui, 2012a).

Hyperkalemia, the most serious symptom of TLS, occurs 6 to 72 hours after the initiation of cytotoxic therapy (McCurdy & Shanholtz, 2012). The rate of onset of the increase in potassium and the presence of renal failure also contributes to the severity of the hyperkalemia (Burghi et al., 2011). Manifestations of hyperkalemia include cardiac arrhythmias, including ventricular ectopy and bradydysrhythmia with peaked T waves, as well as generalized muscle weakness (Clausen, 2010).

Intracellular levels of phosphorus in tumor cells are four times higher than levels found in normal cells (Larson & Pui, 2012a). As the tumor cells lyse after administration of chemotherapy, hyperphosphatemia results and may lead to renal failure and secondary hypocalcemia (Larson & Pui, 2012a). In severe hyperphosphatemia, patients may experience nausea, vomiting, lethargy, and seizures (Cairo & Bishop, 2004). When the calcium phosphorus product is greater than 60 mg^2^/dL^2^, there is a higher risk of calcium phosphate deposition in the renal tubules as well as the heart (Larson & Pui, 2012). This process can lead to renal failure and cardiac arrhythmias (Larson & Pui, 2012a).

The clinical manifestations of hypocalcemia depend on the level of calcium as well as the rate in which the calcium level falls. The symptoms of hypocalcemia are attributed to increased neuromuscular and cardiac excitability and include tetany, muscle spasms, paresthesias, and seizures (Burghi et al., 2011). The cardiovascular manifestations due to hypocalcemia include prolongation of the ST segment, as well as prolongation of the QT interval, which leads to an increased risk of torsade de pointes and sudden death (Burghi et al., 2011).

All of the metabolic disturbances discussed can either exacerbate existing renal insufficiency or be the cause of acute renal insufficiency (ARI). The most common causes of ARI in patients with TLS are hyperuricemia and formation of uric acid crystals in the renal tubules as well as calcium phosphate deposition (Cairo & Bishop, 2004). Acute renal insufficiency can also be related to the nephrotoxicity of certain chemotherapies such as busulfan, bortezomib (Velcade), daunorubicin, and cisplatin (Burghi et al., 2011). It is crucial that renal function and electrolytes are monitored in patients being treated with cytotoxic therapy. In addition, it is very important to begin prophylactic measures to reduce the risk of TLS.

## Prevention

The best treatment for TLS is prevention; correctly identifying those at risk and beginning monitoring are of the utmost importance. Urine output assessment and serum electrolyte screening are the keys to monitoring those at risk for TLS (Larson & Pui, 2012b). Patients at high risk for developing TLS should be assessed for laboratory and clinical signs of the condition every 4 to 6 hours after initiation of chemotherapy. Laboratory monitoring includes evaluation of serum uric acid, phosphate, creatinine, calcium, and LDH levels, in addition to closely monitoring fluid intake and urine output (Coiffier, Altman, Pui, Younes, & Cairo, 2008).

The most important treatment modality in the prevention of TLS is hyperhydration with IV fluids beginning prior to chemotherapy in high-risk patients (Coiffier et al., 2008). Fluid administration increases renal tubular flow and promotes the elimination of urates and phosphates (Coiffier et al., 2008). The goal is to maintain a urine output > 100 mL/hr (Pession et al., 2011). Caution should be taken to avoid fluid overload in patients with preexisting renal insufficiency or cardiac dysfunction. Administration of a loop diuretic may be necessary to maintain adequate urine output in some patients (Larson & Pui, 2012b).

The choice of which IV fluid to administer depends on the clinical situation, but initially 5% dextrose one-quarter normal saline is preferable (Coiffier et al., 2008). Historically, alkalinization to a urine pH of 6.5 to 7.0 with either sodium bicarbonate and/or acetazolamide was standard practice, but the evidence is unclear and controversial (Larson & Pui, 2012b). The rationale behind this practice is to convert uric acid to a more soluble urate salt, which is thought to decrease the risk of uric acid precipitation in the renal tubules (Larson & Pui, 2012b). This standard of care has fallen out of favor as alkalinization of the urine has the potential to promote calcium phosphate deposition in the heart and kidneys (Larson & Pui, 2012b). It is recommended that sodium bicarbonate only be used in patients with metabolic acidosis (Coiffier et al., 2008).

Another important aspect in the prevention of TLS is the use of hypouricemic agents such as allopurinol and rasburicase (Elitek). Allopurinol prevents the formation of new uric acid by blocking the transformation of hypoxanthine and xanthine to uric acid (Kennedy, Koontz, & Rao, 2011). Allopurinol does not reduce the uric acid level before treatment is initiated, and patients with preexisting hyperuricemia should be treated with rasburicase (Larson & Pui, 2012b). Patients at low risk for TLS should receive close monitoring and hydration with or without allopurinol, while patients at intermediate risk for TLS would be candidates for hydration plus allopurinol prophylaxis (Cairo et al., 2010). Allopurinol prophylaxis should begin up to 3 days before initiation of chemotherapy (Kennedy, Koontz, & Rao, 2011).

On the other hand, rasburicase is a recombinant urate oxidase enzyme that causes rapid reduction in uric acid levels by converting uric acid to a more water-soluble compound called allantoin (Coiffier et al., 2008). Although allopurinol prevents the formation of uric acid, rasburicase lowers already high levels of uric acid in addition to preventing further hyperuricemia (Larson & Pui, 2012b). In contrast with patients at low risk for developing TLS, patients at high risk should receive increased hydration (3 L/m^2^/day) and rasburicase for one dose, repeated if necessary (Cairo et al., 2010). For adult patients at intermediate risk for TLS, electrolytes should be monitored every 8 hours after initiation of chemotherapy and for at least 24 hours after completion of chemotherapy (Larson & Pui, 2012b).

## Treatment

Regardless of appropriate prophylactic measures, about 3% to 5% of patients receiving chemotherapy will develop laboratory and/or clinical TLS (Larson & Pui, 2012b). Patients with TLS should generally receive continuous cardiac monitoring as well as measurement of electrolytes and uric acid every 4 to 6 hours (Larson & Pui, 2012b). Treatment is aimed at reversing the electrolyte derangements as well as treating the acute kidney injury. All patients with laboratory and clinical TLS should receive hydration via a central venous catheter as well as rasburicase (Tosi et al., 2008). Rasburicase is administered at 0.20 mg/kg/day for up to 5 days (Kennedy, Koontz, & Rao, 2011). It is contraindicated in patients with a history of glucose-6 phosphate dehydrogenase deficiency, as it will cause hemolysis in that patient population; allopurinol should be substituted (Cairo et al., 2010). The recommended daily dose of allopurinol is 300 mg in patients with a glomerular filtration rate > 50 mL/min (Kennedy, Koontz, & Rao, 2011).

Hyperkalemia is the most concerning electrolyte abnormality as it can cause cardiac arrhythmias and sudden death. Hyperkalemia is typically defined as a serum potassium level ≥ 6 mmol/L (Cairo & Bishop, 2004). Mild hyperkalemia (< 6 mmol/L) can be corrected with IV hydration, loop diuretics, and sodium polystyrene (Tosi et al., 2008). In the treatment of severe hyperkalemia, IV injection of insulin and dextrose will achieve immediate, yet temporary, reduction in serum potassium levels by shifting extracellular potassium into the intracellular space (Lehnhardt & Kemper, 2011). Careful EKG monitoring should also be completed in hyperkalemic patients (Tosi et al., 2008). Additionally, patients should be instructed to limit intake of potassium-rich foods, and supplemental potassium should be avoided (Cairo & Bishop, 2004). If necessary, hemodialysis can remove potassium successfully in patients with symptomatic or severe hyperkalemia. Continuous renal replacement therapy may be needed to avoid rebound hyperkalemia in patients with TLS (Wilson & Berns, 2012).

Hyperphosphatemia is an additional electrolyte disturbance experienced in TLS that has the potential to cause acute kidney injury. Mild hyperphosphatemia (< 1.62 mmol/L) does not require treatment or can be treated with aluminum hydroxide orally or via nasograstric tube (Tosi et al., 2008). Lowering moderately high serum phosphate levels (> 2.1 mmol/L) can be achieved by administering aggressive IV hydration in addition to phosphate binder therapy (Larson & Pui, 2012b). Some patients may require continuous hemodialysis to correct severe hyperphosphatemia (Cairo & Bishop, 2004). At times, secondary hypocalcemia can occur as a result of hyperphosphatemia. Since the risk of calcium phosphate deposition is high, only patients with symptomatic hypocalcemia require treatment with IV calcium gluconate (Cairo & Bishop, 2004).

Finally, severe hyperuricemia can cause acute obstructive uropathy. As mentioned previously, patients who present with LTLS or CTLS would be candidates for rasburicase administration as prophylaxis or treatment for hyperuricemia (Cairo & Bishop, 2004). All of the previously mentioned electrolyte abnormalities can result in acute kidney injury. The cause of acute kidney injury can be secondary to hyperuricemia or calcium phosphate deposition, leading to obstructive uropathy syndrome (Cairo & Bishop, 2004). The mainstay of renal dysfunction management in TLS is strict monitoring of fluid intake and output as well as electrolyte and hypertension management (Cairo & Bishop, 2004). It is also critically important to manage hyperuricemia and hyperphosphatemia to avoid further uric acid crystallization and calcium phosphate deposition (Cairo & Bishop, 2004). Despite these approaches, some patients may require hemodialysis or continuous arteriovenous hemofiltration (Cairo & Bishop, 2004).

## Summary

Tumor lysis syndrome is a composition of metabolic abnormalities that are a result of purine catabolism and release of electrolytes from lysis of tumor cells following cytotoxic therapy. Hematologic malignancies are at highest risk of causing TLS, as well as bulky tumors with high response to chemotherapy and/or radiation therapy. Prevention of TLS is of the utmost importance. Patients should be identified early on as being at low, intermediate, or high risk of developing TLS so that prophylactic measures can be taken. Quick identification and correction of the metabolic derangements associated with TLS is necessary to avoid life-threatening complications such as renal failure, cardiac dysrhythmias, seizures, and even death due to multiorgan failure.

## Acknowledgment

The author would like to give special thanks to Cheryl Tompkins, MSN, CRNP, AOCNP^®^, for her assistance with the preparation of this article.
